# Supplementary material for: ERECTA genes and their ligands regulate shoot and inflorescence architecture in maize
Source: Nat Commun. 2026 Jan 12;17:110. doi: 10.1038/s41467-025-67634-3 (PMC12795852; doi:10.1038/s41467-025-67634-3)
Supplement: Supplementary file 1 — Supplementary Information [file 41467_2025_67634_MOESM1_ESM.pdf]

# *ERECTA* genes and their ligands regulate shoot and inflorescence architecture in maize

Liu *et al.*

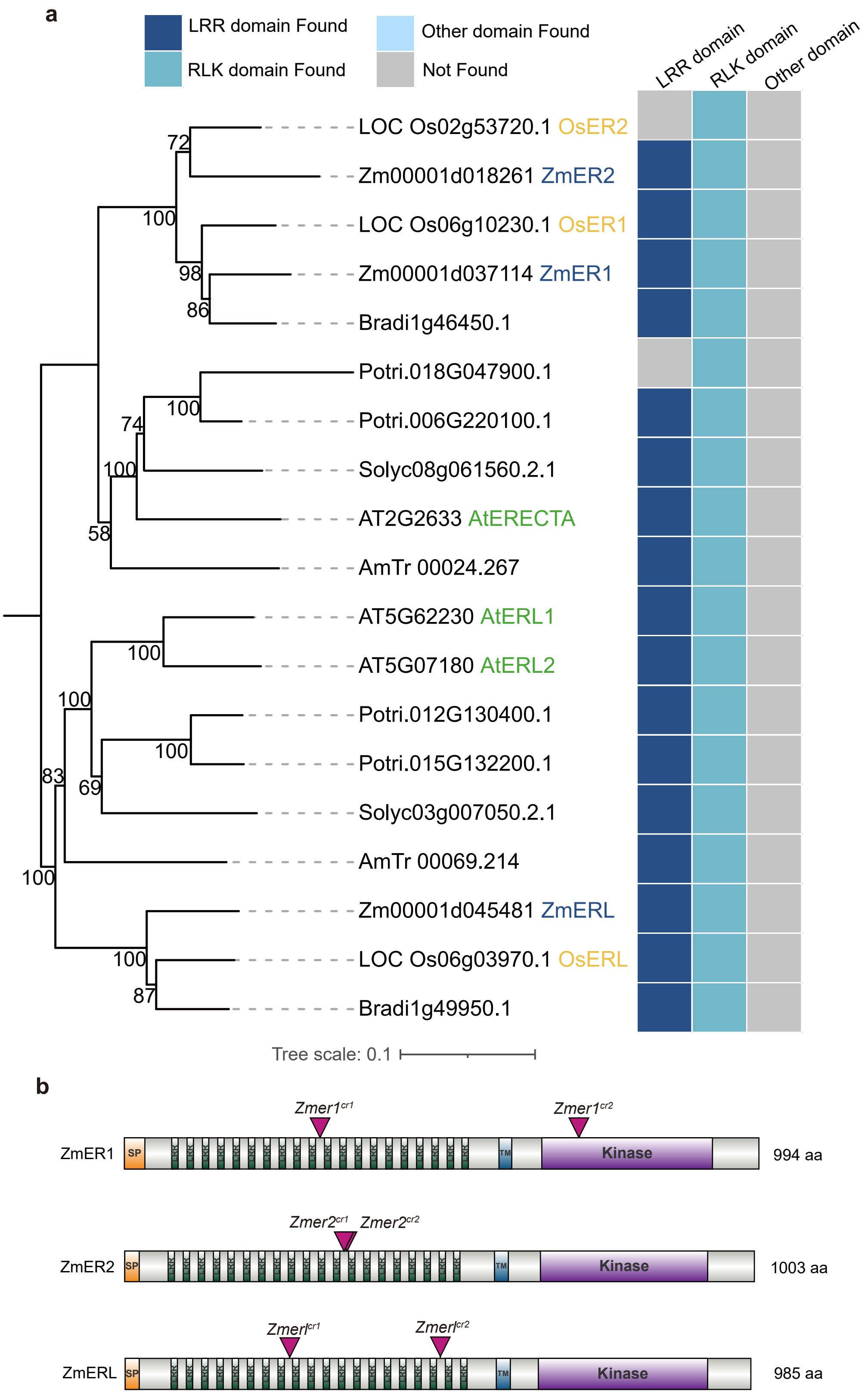


**Supplementary Fig. 1. ZmER phylogenetic tree and domains. a** Phylogenetic analysis reveals that the arabidopsis *ER* has two orthologs genes in maize, *ZmER1* and *ZmER2*, while *AtERL1* and *AtERL2* correspond to a single ortholog, *ZmERL* in maize. The rice *OsER1*, *OsER2* and *OsERL* are also included in the tree. **b** Schematic representation of the maize ER protein structure with CRISPR/Cas9 gRNA target sites shows by inverted triangles.

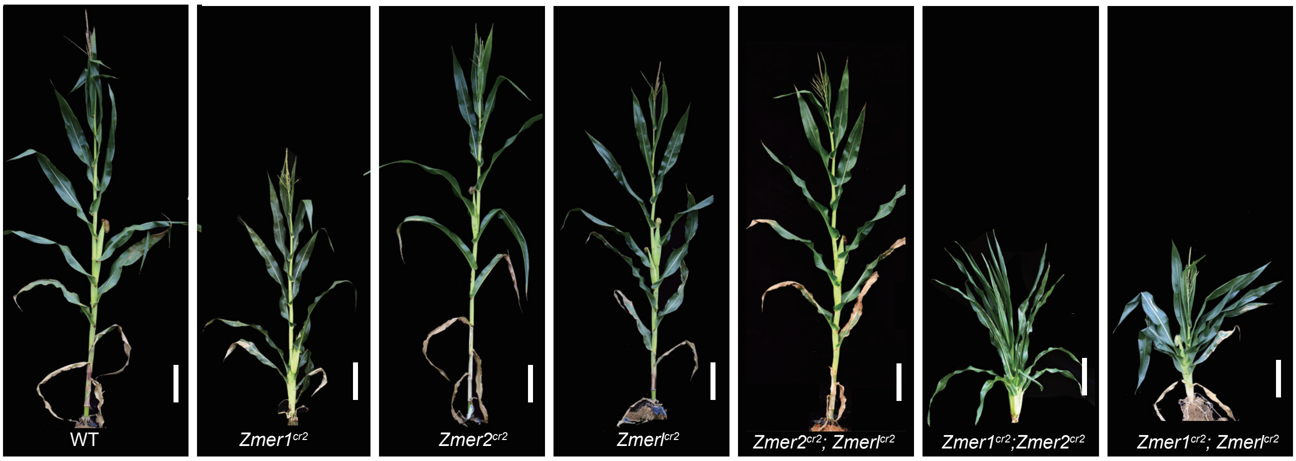


**Supplementary Fig. 2. The phenotypes of the second alleles of different *Zmer* mutants.** The *Zmer1*$^{cr2}$ single mutants, *Zmer1*$^{cr2}$;*Zmer2*$^{cr2}$ double mutants, and *Zmer1*$^{cr2}$;*Zmerl*$^{cr2}$ double mutants exhibit reduced stature compared to wild-type plants. Scale bars, 20 cm.

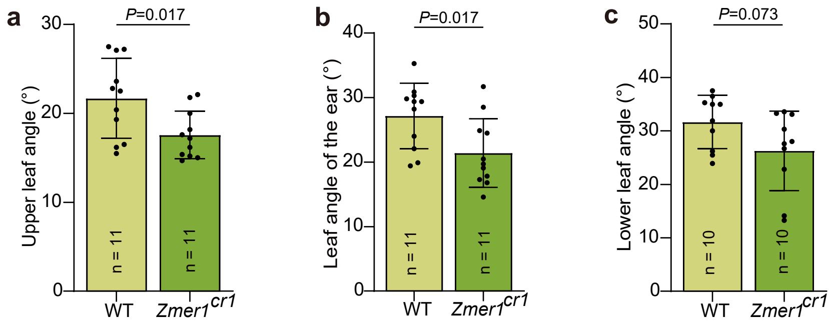


**Supplementary Fig. 3. *Zmer1$^{cr1}$* mutants exhibit significantly reduced leaf angles. a-c** Statistical analysis reveals the leaf angles at three different positions of *Zmer1$^{cr1}$* mutants are reduced compared to those in the wild type. Upper leaf angle, angle between midrib and upper stem of 1st leaf above ear node; Leaf angle of the ear, angle between midrib and ear leaf; Lower leaf angle, angle between midrib and lower stem of 1st leaf above ear node. The sample size (n) and *p*-values for each group are labelled in the figure, *p*-values are calculated using two-tailed Student's *t*-tests. Error bars represent mean ± s.d.

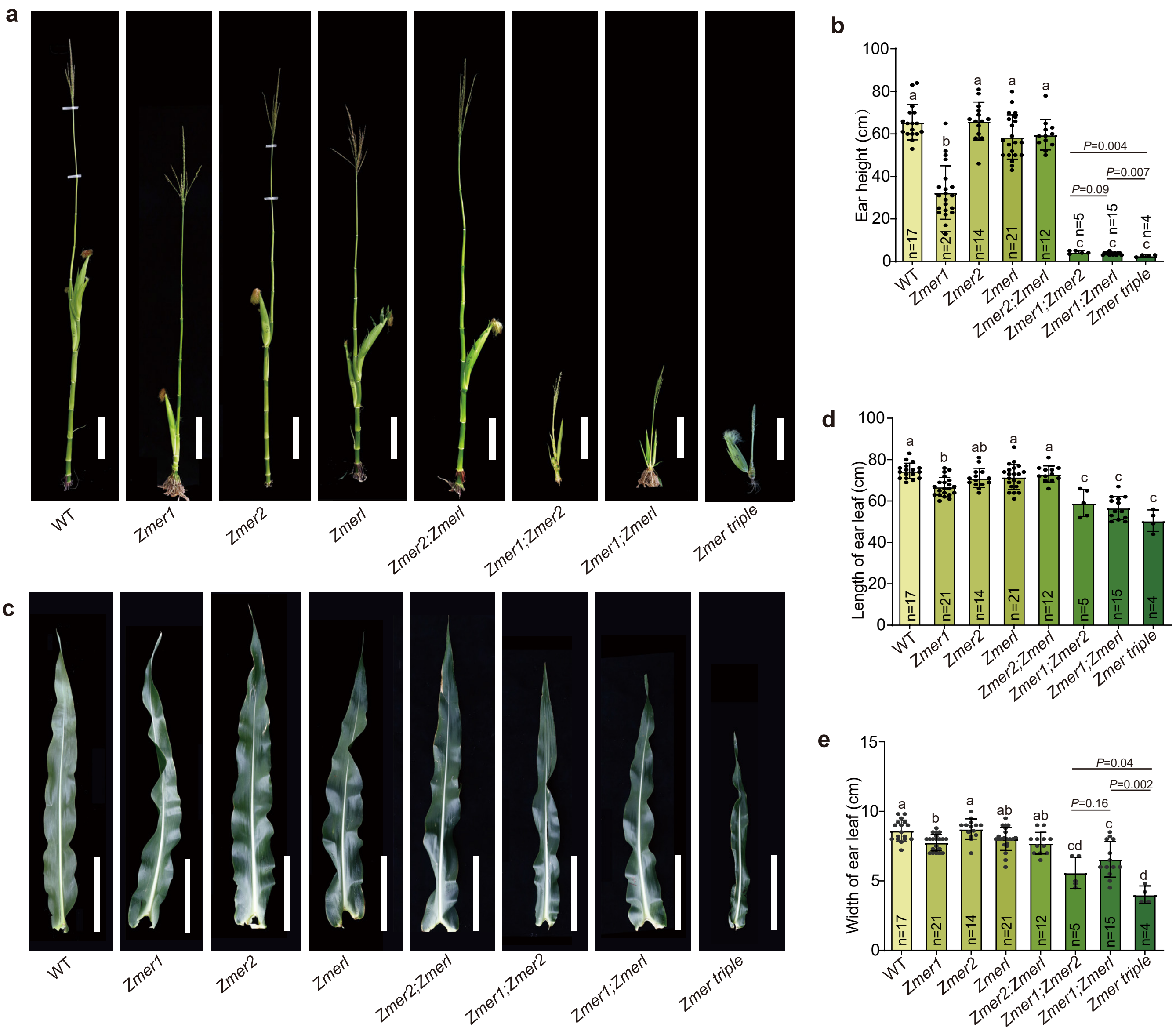


**Supplementary Fig. 4. The internode and leaf phenotypes of *Zmer* mutants**. **a** The *Zmer1* single mutants, *Zmer1;Zmer2* and *Zmer1;Zmerl* double mutants, as well as the *Zmer* triple mutants, have lower ear height. **b** Statistic analysis of ear height in WT and various *Zmer* mutants. Different letters indicate statistical significance groups at $p < 0.05$ (one-way ANOVA with post hoc Tukey's multiple comparison test). Although the *Zmer1;Zmer2*, *Zmer1;Zmerl* and the triple mutants are classified within the same group, the ear height of the triple mutant is significantly lower than that of both double mutants as determined by two-sided unpaired Student's *t*-test. The sample size (n) and *p*-values for each group are labelled in the figure. **c** Representative images of ear leaves in WT and *Zmer* mutant plants. **d**-**e** Statistic analysis of the ear leaf length (**d**) and ear leaf width (**e**) in WT and various *Zmer* mutants. Different letters indicate significantly distinct groups as determined by one-way ANOVA. Although the *Zmer1;Zmer2* and the triple mutants are classified within the same group by one-way ANOVA, the leaf width of the triple mutant is significantly smaller than the double mutants as determined by two-sided unpaired Student's *t*-test.The sample size (n) and p-values for each group are labelled in the figure. Scale bars. 20 cm (**a**,**c**). For **a-e**, phenotypic analyses used the *cr1* alleles for all three genes. Superscript "cr1" are omitted for space. The source data underlying the statistical analysis in **b**, **d** and **e** are provided in the Source Data file.

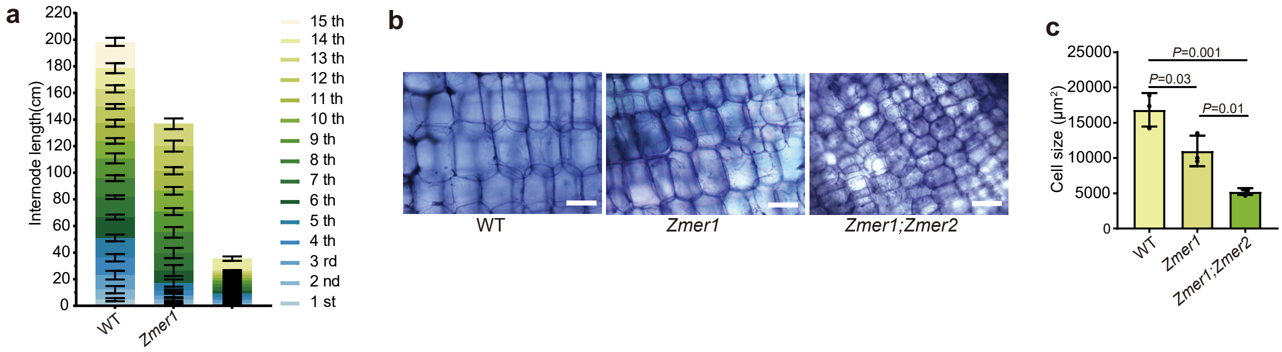


**Supplementary Fig. 5. The internode length and cell size are reduced in *Zmer1* and *Zmer1;Zmer2*. a** Measurement of internode length in mature WT, *Zmer1*, and *Zmer1;Zmer2* plants. Internodes are counted from the base, with the lowest internode designated as the first. Different internodes are indicated with different colors. Error bars represent standard deviation, n≥7. **b** Longitudinal sections of the third internode below the ear at the 12-leaf stage in WT, *Zmer1*, and *Zmer1;Zmer2* plants. Scale bars: 50 μm. **c** Quantification of cell size in WT, *Zmer1*, and *Zmer1;Zmer2*. Data from each genotype are collected three groups, with each group consisting of at least ten fields of view. *p*-values are calculated using a two-tailed Student's *t*-test. Error bars represent mean ± s.d.

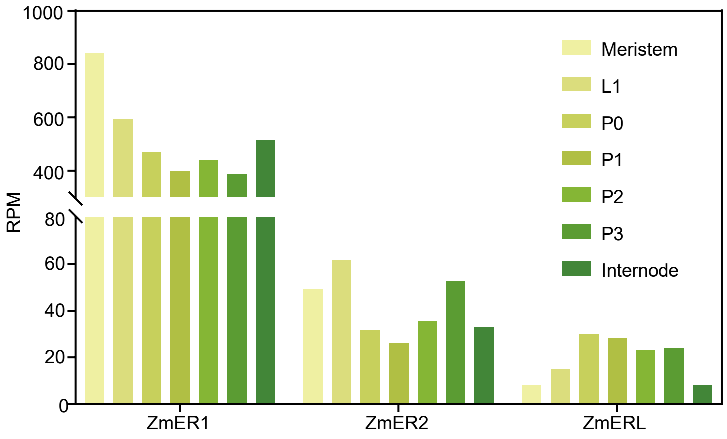


**Supplementary Fig. 6**. **The expression pattern of *ZmER1, ZmER2*, and *ZmERL* in SAM*.*** *ZmER1*, *ZmER2* and *ZmERL* are expressed in the shoot apical meristem (SAM), including the meristem, epidermal layer of dividing cells (L1), the incipient primoridium within the SAM (P0), the first visible leaf is a plastochron 1 (P1), plastochron 2 (P2), plastochron 3 (P3) and internodes. The expression levels of *ZmER1* are significantly higher than *ZmER2* and *ZmERL* in different meristem tissues.

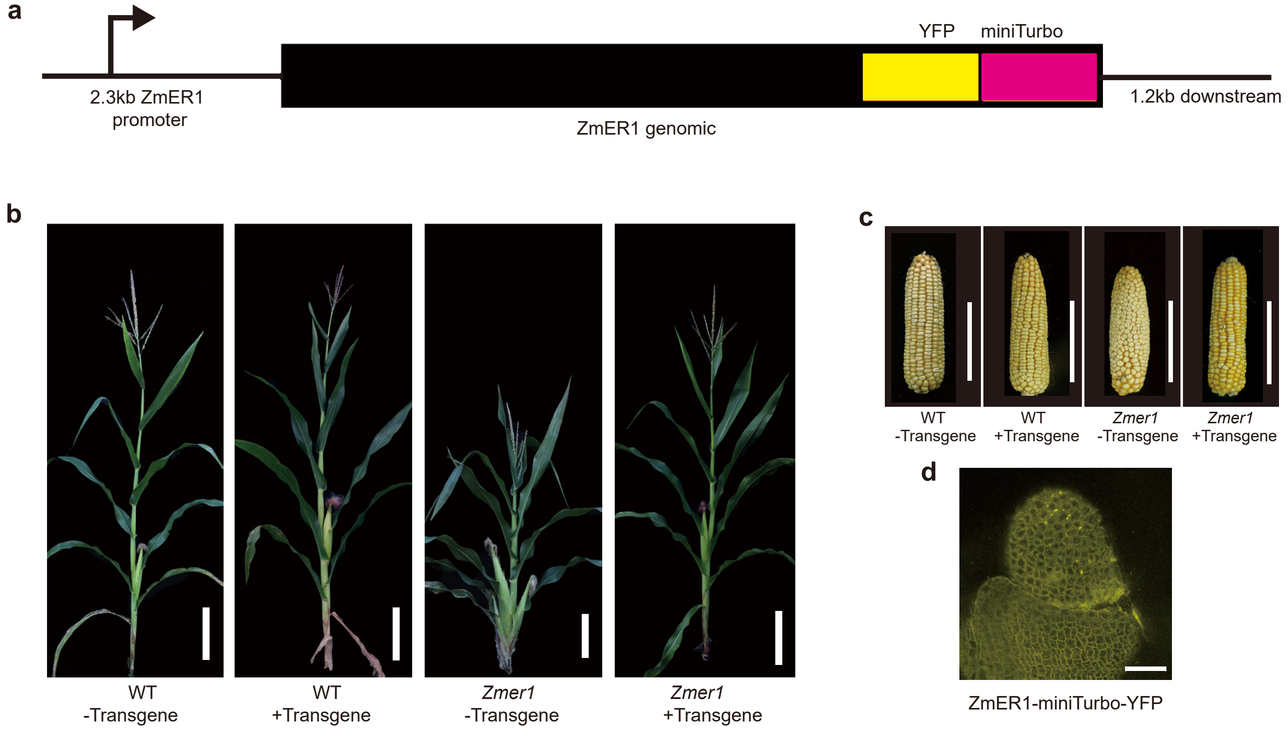


**Supplementary Fig. 7. Native transgene expression of *ZmER1* rescues the stature and ear phenotypes of the mutants. a** The miniTurbo-YFP is fused with ZmER1 genomic sequence at the C-terminus driven by a 2.3-kb native upstream sequence and followed by a 1.2-kb downstream sequence**. b-c** The *ZmER1* transgene successfully rescues the dwarf and fasciated ear phenotypes of the *Zmer1* mutant. **d** ZmER1-miniTurbo-YFP is expressed throughout the SAM, and the protein is primarily localized to the membrane. Each experiment is independently repeated three times with similar results. Scale bars, 20 cm (**b**),10 cm (**c**) and 50 µm (**d**).

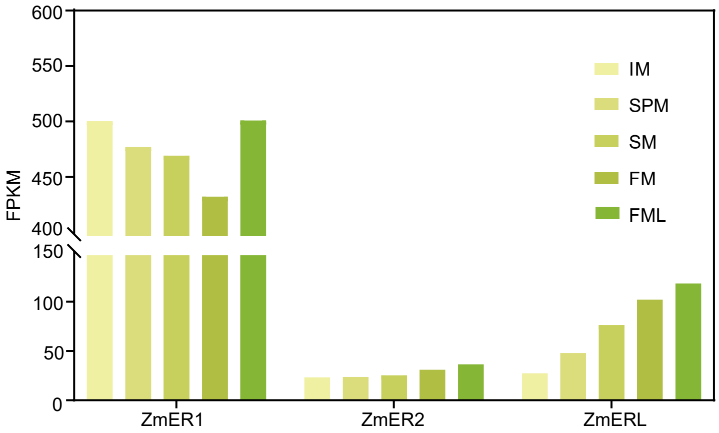


**Supplementary Fig. 8. The expression pattern of *ZmER1, ZmER2*, and *ZmERL* in IM.** *ZmER1, ZmER2*, and *ZmERL* are expressed in the inflorescence meristem (IM), spikelet pair meristem (SPM), spikelet meristem (SM), and floral meristem (FM).

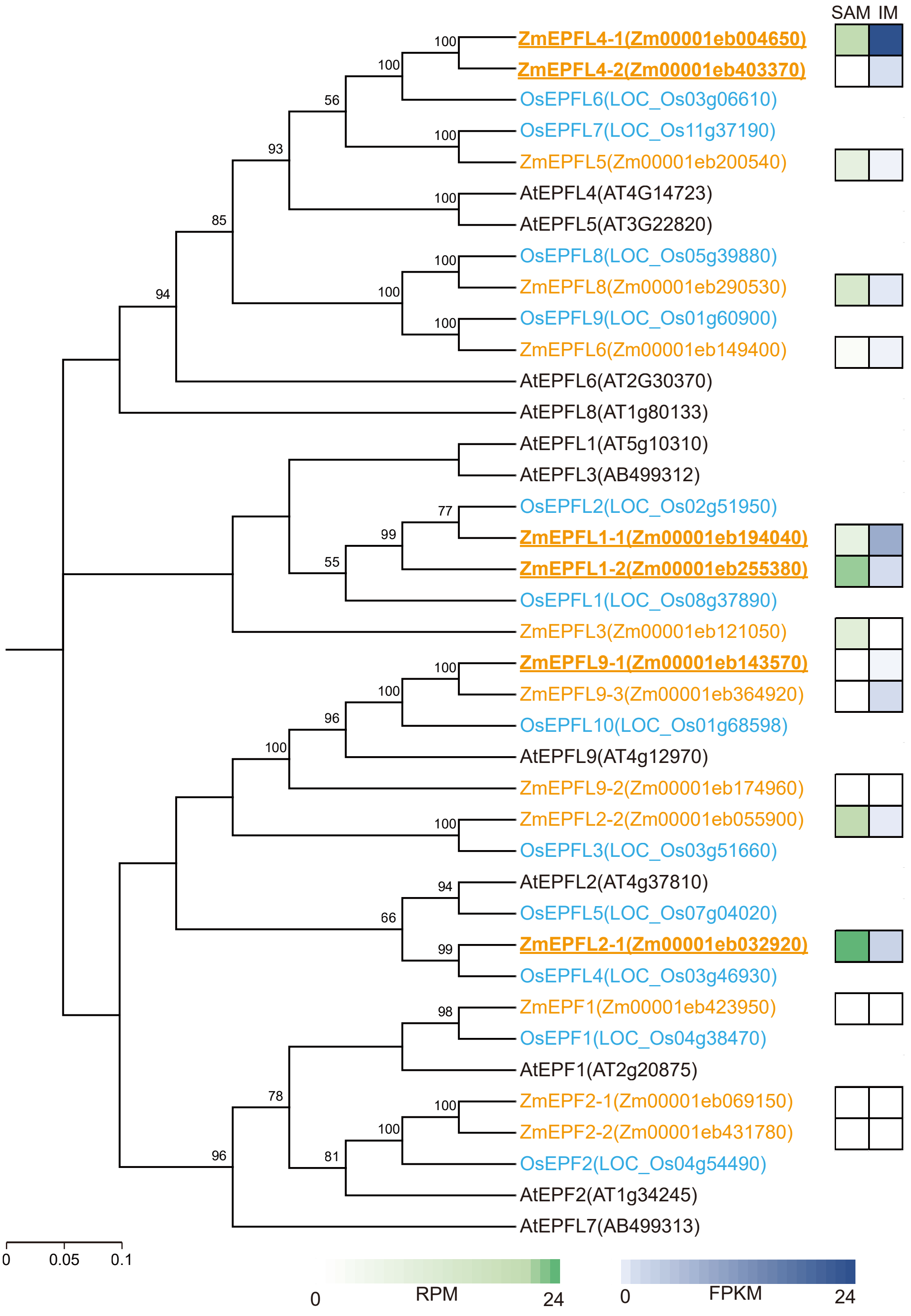


**Supplementary Fig.9. Phylogenetic analysis of the EPF/EPFL gene family in maize (orange), rice (blue), and arabidopsis (black).** The six genes selected for CRISPR-mediated editing are highlighted in bold orange and underlined. The right panel represents the relative expression patterns of maize EPF/EPFL family genes in the SAM and IM, based on RPM and FPKM values from publicly available RNA-seq datasets in NCBI (SRP101301 and PRJNA911902).

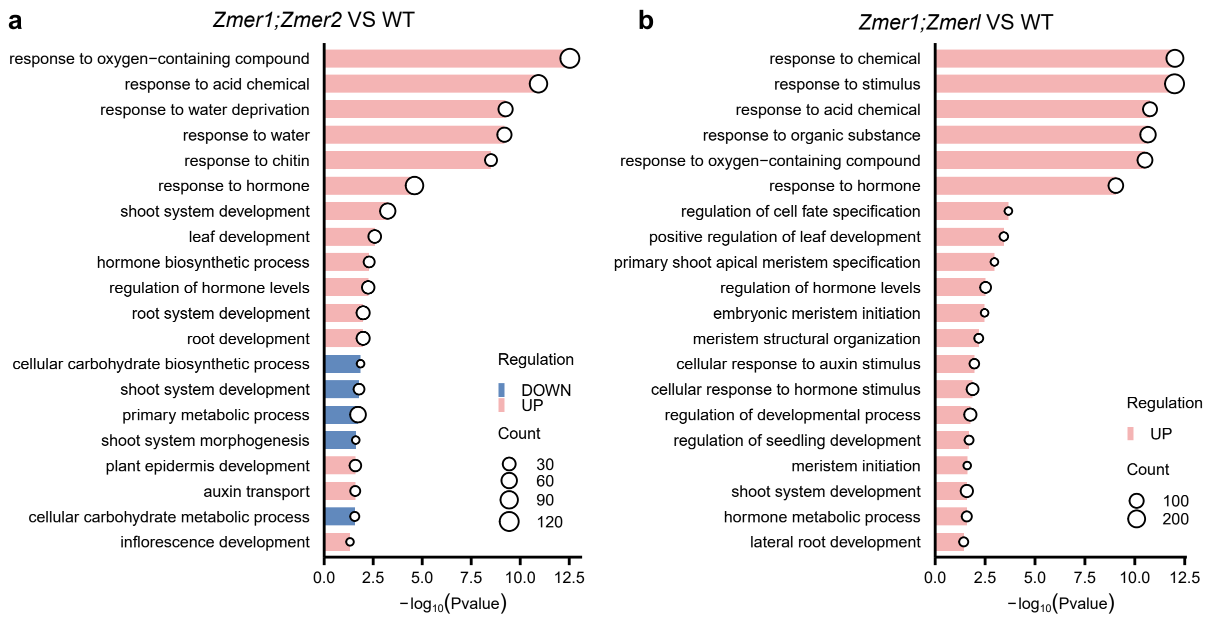


**Supplementary Fig. 10. Gene Ontology (GO) enrichment analysis of differentially expressed genes in *Zmer1;Zmer2* and *Zmer1;Zmerl*. a** In *Zmer1;Zmer2*, upregulated and downregulated genes are significantly enriched in multiple biological themes, including response to hormone, regulation of hormone levels and inflorescence development. **b** In *Zmer1;Zmerl*, upregulated genes are significantly enriched in biological themes such as response to hormone and regulation of cell fate specification and no significant enrichment is detected among down-regulated genes. Bar plots show the significance of enriched GO biological process (BP) terms ($-\log_{10}$(*p*-value)), with circle sizes indicating the number of associated genes.

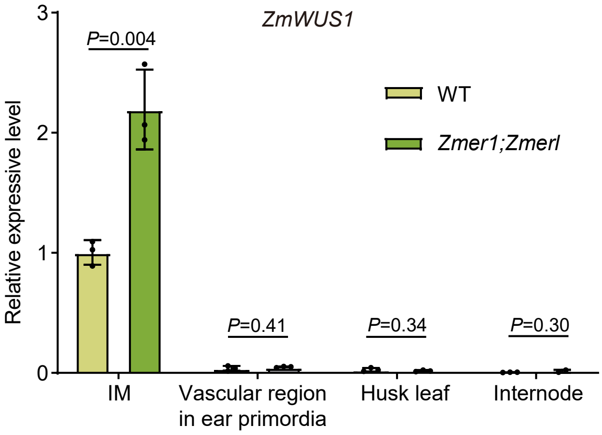


**Supplementary Fig. 11. Relative expression levels of *ZmWUS1* in WT and *Zmer1;Zmerl* plants in different tissues.** In the inflorescence meristem (IM), ZmWUS1 expression is significantly higher in *Zmer1;Zmerl* compared with WT. In contrast, expression is barely detectable in vascular tissues in ear primordia, young husk leaf, and internodes, with no significant difference between genotypes. Data are presented as mean ± s.d., *p*-values are calculated using a two-tailed Student's *t*-test, RT-qPCR is performed with one biological replicate and three technical replicates, approximately 10 IMs are used in each biological replicate.

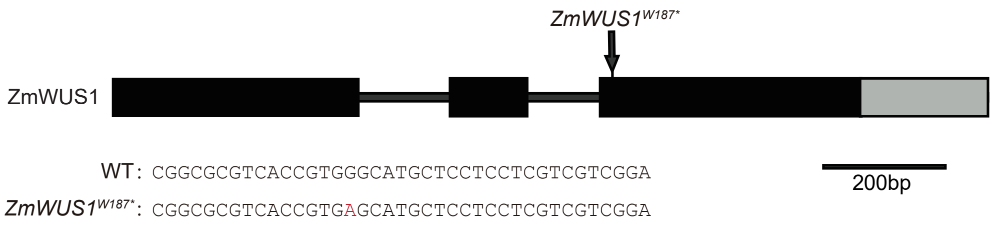


**Supplementary Fig. 12. Identification of the *Zmwus1* mutant.** The *ZmWUS1*$^{W187*}$ mutant harbors an EMS-induced G-A transition, which causes a premature stop codon.

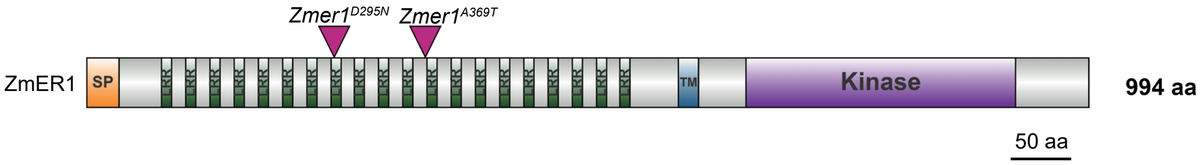


| ID | Allele | Mutant ID | Codon | Effect |
|---|---|---|---|---|
| 1 | *Zmer1* $^{D295N}$ | EMS4-13a82f | Gat/Aat | D295N |
| 2 | *Zmer1* $^{A369T}$ | EMS4-089b7b | Gcg/Acg | A369T |

**Supplementary Fig. 13. Identification of two nonsynonymous alleles of *Zmer1*.** Inverted triangles indicate the positions of the two nonsynonymous alleles and the detailed information are listed in the table below.

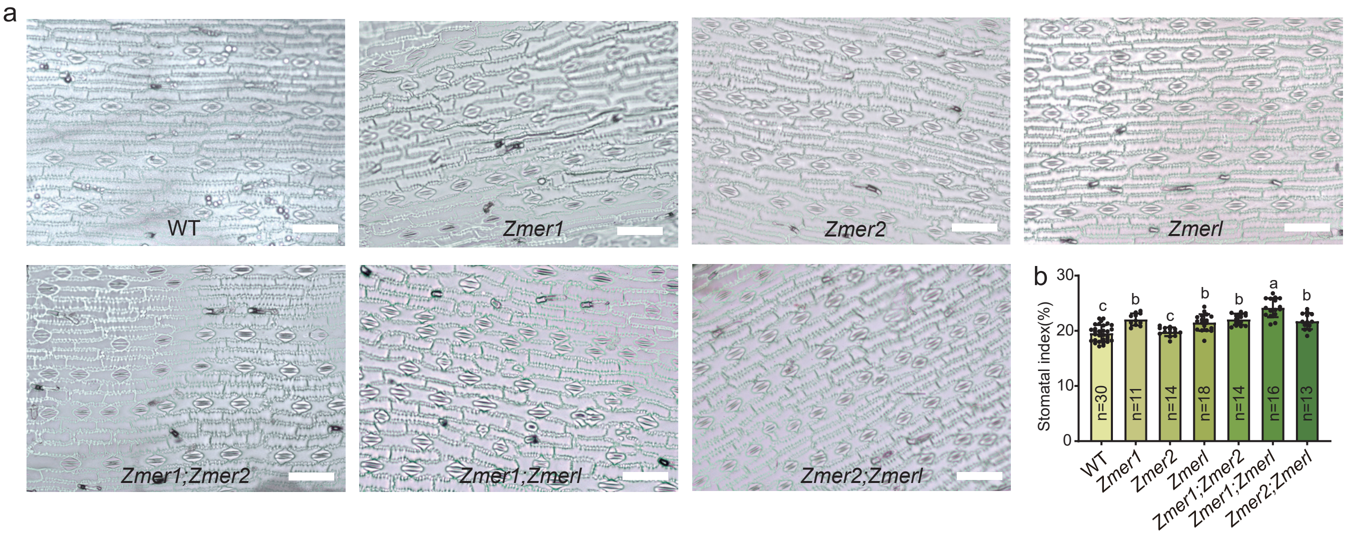


**Supplementary Fig. 14**. ***ZmER* genes affect stomatal development**. **a** Representative image of abaxial epidermal showing stomatal patterning in *Zmer* single and double mutant. **b**. Quantification of the stomatal index for the indicated genotypes. Except for the *Zmer2* single mutant, all other single and double mutants display a significantly higher stomatal index, with the *Zmer1;Zmerl* double mutant showing the largest increase. Bars represent mean ± s.d. (n>10). Different letters indicate significantly distinct groups as determined by one-way ANOVA. Scale bar, 100 μm.

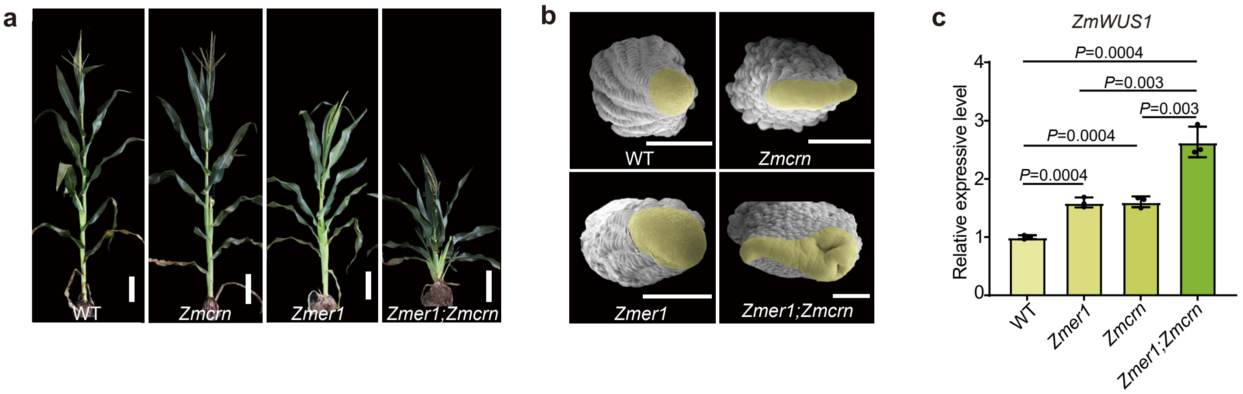


**Supplementary Fig. 15. The double mutant analysis of *Zmer1* and *Zmcrn* in maize. a** *Zmer1;Zmcrn* double mutants are shorter compared to *Zmer1* single mutants. **b** Scanning electron microscopy (SEM) images of ear primordia from WT, *Zmer1, Zmcrn* and *Zmer1;Zmcrn* reveal that inflorescence meristems are more fasciated in the double mutants than in the single mutants. Scale bars, 20 cm (**a**), 500 μm (**b**). Each experiment is independently repeated three times, yielding consistent results. **c** Relative transcriptional level of *ZmWUS1* in WT, *Zmer1, Zmcrn* and *Zmer1;Zmcrn* determined by RT-qPCR. Data are presented as mean values ±s.d., *p*-values are calculated using a two-tailed Student's *t*-test, RT-qPCR is performed with one biological replicate and three technical replicates, approximately 10 IMs are used in each biological replicate.
