# Supplementary material for: ERECTA genes and their ligands regulate shoot and inflorescence architecture in maize
Source: Nat Commun. 2026 Jan 12;17:110. doi: 10.1038/s41467-025-67634-3 (PMC12795852; doi:10.1038/s41467-025-67634-3)
Supplement: Supplementary file 2 — Description of Additional Supplementary Files [file 41467_2025_67634_MOESM2_ESM.pdf]

Description of Additional Supplementary Files

File Name: Supplementary Data 1

Description: Primers used in this study.

File Name: Supplementary Data 2

Description: Differentially expressed genes in *Zmer1;Zmer2* double mutants compared to WT.

File Name: Supplementary Data 3

Description: Differentially expressed genes in *Zmer1;Zmerl* double mutants compared to WT.
